# Strategies for Genomic Medicine Education in Low- and Middle-Income Countries

**DOI:** 10.3389/fgene.2019.00944

**Published:** 2019-10-08

**Authors:** Nirmala D. Sirisena, Vajira H. W. Dissanayake

**Affiliations:** Human Genetics Unit, Faculty of Medicine, University of Colombo, Colombo, Sri Lanka

**Keywords:** developing world, genomic education, genomic literacy, genomic medicine, healthcare

## Introduction

Implementing genetic and genomic medicine is dependent to a large extent on the successful training of a genomics workforce with expertise in interpreting, communicating, and integrating genomic information in a clinical setting. In order to effectively implement genomic medicine at different levels of healthcare delivery, strategies for establishing training in core competencies of genetics and genomics targeted at the undergraduate, postgraduate, and continuing professional development levels need to be in place. Several approaches have been adopted in Western countries like the UK and USA to ensure that their healthcare workforce is adequately trained and competent to effectively use genetic and genomic information in their professional practice. However, this is not the case in most low- and middle-income countries (LMICs) located in regions of East Asia and the Pacific, Central and South Asia, Latin America, and the Caribbean, North and Sub-Saharan Africa, with a gross national income of $1,026–$3,995. In many of these countries, this necessity has been plagued by numerous challenges stemming from the lack of local capacity to plan and carry out the required training of the healthcare workforce. The other contributory factors are the scarcity of adequate funding for training as well as establishing core facilities needed for delivering these services around which such training programs could be implemented and delivered (Sirisena and Dissanayake, 2018). Herein, we provide a concise overview of the various challenges faced in achieving genomic literacy for integrating genomic medicine into the healthcare setting in LMICs and potential strategies for overcoming such limitations.

## Situation Analysis Of The Challenges

Several initiatives have promoted genomic research and infrastructure and capacity development in many LMICs such as the Human Heredity and Health in Africa (H3Africa), the Qatar Genome Project, the Mexico National Institute of Genomic Medicine (INMEGEN), and the Collaborative African Genomics Network (CAfGEN) (Tekola-Ayele and Rotimi, 2015; Mlotshwa et al., 2017; Mboowa and Sserwadda, 2019). As genomic technologies rapidly advance and genomic sequencing becomes increasingly affordable, even in the LMICs, immense volumes of genomic data are generated with potential for guiding clinical decision making in the healthcare setting. However, the advent of such clinically actionable genomic information creates a dilemma as most healthcare providers in these countries are not competent in interpreting and communicating these results due to inadequate genomics knowledge and skills, thereby depriving patients from making informed decisions regarding personalized, targeted disease screening, prevention, diagnostics, and treatment approaches that can influence health and disease management (Metcalfe et al., 2002; Guttmacher et al., 2007; Cohn et al., 2015; Mboowa and Sserwadda, 2019). Thus, in the current genomic era, it is vital that LMICs take necessary measures to educate and build up a healthcare workforce that is effectively trained to integrate genetic and genomic information into their clinical practice (de Abrew et al., 2014; Sirisena et al., 2016a). However, the practical challenges for implementing such educational initiatives are quite diverse (Guttmacher et al., 2007; de Abrew et al., 2014; Sirisena et al., 2016b; Sirisena and Dissanayake, 2018). Some of them are addressed below.

A major impediment in most LMICs is the lack of personnel trained in genetics, genomics, and bioinformatics who could serve as a core team to plan and develop training programs and clinical and laboratory facilities around which such programs could be implemented and delivered (Wonkam et al., 2010; de Abrew et al., 2014). The lack of adequate infrastructure such as cytogenetic and molecular genetic laboratories and tools for providing quality training and services is a huge setback. Disparities in health priorities in most LMICs is an important factor contributing to lack of sufficient funding for developing, implementing, and sustaining genomic-based educational initiatives (Sirisena and Dissanayake, 2018). Consequentially, this has led to the slow pace of translation of genomics research from the bench to the bedside resulting in a lack of perception of the clinical relevance of genomics and its clinical utility and potential benefit for improving health-related patient outcomes (Tekola-Ayele and Rotimi, 2015).

Lack of access to genomic-based educational resources and e-learning tools in local languages for training at the secondary, tertiary, and continuing professional development levels, lack of access to internet facilities and/or the skills and confidence to use web-based learning resources by some healthcare providers are further deterrents (Mitropoulos et al., 2015). Another limiting factor is the time constraints of busy healthcare providers who find it difficult to keep up with the rapid pace of clinical genomic advances and thereby tend to pursue only those educational opportunities that cater for the immediate needs of their patients (Skirton et al., 2010; de Abrew et al., 2014; Tekola-Ayele and Rotimi, 2015).

Additional challenges include institutional matters and differences in the educational systems across the LMICs, such as the structure and sequence of existing undergraduate medical curricula resulting in significant differences in the content and delivery of genomic education. Misconceptions and flawed assumptions among medical students and health professionals that diseases fall strictly into genetic and non-genetic categories rather than into a continuum of interaction between genetic and non-genetic components are other limiting factors (Skirton et al., 2010; de Abrew et al., 2014; Tekola-Ayele and Rotimi, 2015).

## Strategies And Way Forward

Genomics‐related educational initiatives to improve the genetic and genomic literacy among healthcare professionals in LMICs would require a multi-faceted approach, depending on the national priorities and the financial capabilities of each country. The pre-requisites needed for the development of genetic and genomics literacy include the following: recognition of the need, definition of the knowledge and skills required, development and implementation of educational initiatives and evaluation to assess the achievement of the desired outcomes (Gaff et al., 2007; Thurston et al., 2007; Skirton et al., 2010; de Abrew et al., 2014). Some of the core areas in which healthcare professionals need to develop competency in include: genetic variation in health and disease, the role of the family history in determining the modes of inheritance of genetic disorders and assessment of genetic risk, indications for referral for genetic evaluation and testing, assessing the clinical validity and utility of genetic testing for specific clinical conditions, ordering and interpreting genetic and genomic tests, communicating genomic information effectively, genetic counselling and facilitating informed decision making by patients, integrating genetic information into clinical management decisions, and the complex ethical and psychosocial issues related to genetics and genomics (Guttmacher et al., 2007; Telner et al., 2008; Korf, 2013). Educational programs on genetics and genomics should ideally incorporate a pre-service education component for those in training prior to their onset of clinical practice as well as a continuing education component along with professional practice guidelines to cater for those currently in clinical practice (de Abrew et al., 2014; Korf et al., 2014; Manolio et al., 2015). Five entrustable professional activities (EPAs) that encompass a basic set of genomic skills with clinical applications across different levels of healthcare and between medical specialties have been identified. They include: family history, genomic testing, genomic-guided therapeutics, somatic cancer genomics, and microbial genomic information (Korf et al., 2014; Institute of Medicine, 2015).

Education and training in the basics of genomics and bioinformatics could be introduced at the level of pre-undergraduate education while more advanced training could be at the undergraduate and postgraduate levels. It is also necessary to address any misconceptions among medical students and health professionals and create awareness that genomics underlies the whole of pathophysiology and constitutes the fundamental science of health and disease and should therefore not be treated solely as a medical specialty having implications for only a few areas of clinical practice (Guttmacher et al., 2007).

Even though basic genetics content focusing mainly on the rare Mendelian disorders is integrated into the basic sciences courses of most medical undergraduate curricula in varying depths and durations, there is a need for it to be applied across the entire curriculum, ending with real life patients during clinical training through inclusion of case-based clinical examples to illustrate the genetic and genomic determinants and mechanisms underlying common complex diseases such as heart disease, hypertension, diabetes mellitus, etc. (Korf, 2002; Guttmacher et al., 2007; Telner et al., 2008). Such approaches would facilitate bridging the gap between the basic sciences and the clinical training and instill in the trainee the perception that genetics and genomics is clinically relevant. In situations where such integration is non-existent, revision of the undergraduate medical curricula to incorporate genetics and genomics modules tailor-made towards disease conditions that are relevant in the local context is warranted (Mboowa and Sserwadda, 2019).

At the postgraduate level, training in genetics and genomics should be incorporated into both specialty and sub-specialty training programs. It is also important that examinations for licensure and certification should include a substantial number of genetics-related questions. Additional strategies for building bridges between genetics and genomics and other specialties include the establishment of joint specialty training programs that combine medical genetics and genomics with another major discipline and the development of subspecialty training for individuals trained outside of medical genetics and genomics and introduction of new graduate programs in genetics and genomics (Mboowa and Sserwadda, 2019; Sirisena and Dissanayake, 2019). Thus, it would be necessary for the largely public funded academic institutions in LMICs to take the necessary steps to request for increased allocation of funds from national budgets for the realization of the above outcomes (Sirisena and Dissanayake, 2017).

In order to cater for the needs of healthcare professionals currently in clinical practice who have not received training in basic genetics content during their undergraduate medical training, continuing professional development programs incorporating basic educational materials should be introduced by hospitals and health systems, professional medical associations and societies to equip clinicians to provide some genetic services on their own, while providing clear guidelines for referral to genetics specialists when necessary (Houwink et al., 2011; Sirisena and Dissanayake, 2019). Specialized training such as genomics workshops, seminars, postgraduate courses, and massive open online courses also provide opportunities for clinicians to obtain up-to-date information with the purpose of improving genetics and genomics knowledge, attitudes, and skills in a cost-effective and time-efficient manner (Sirisena et al., 2016a; Mboowa and Sserwadda, 2019). Many organizations have developed or are in the process of developing such point-of-care, electronic decision-support systems and continuing professional development courses for healthcare providers based on the best practices in adult learning, such as interactivity, case-based learning, and skill-focused objectives (Reed et al., 2016). A needs-driven, learner-centric, evidence-based, outcomes-oriented, and practice-embedded continuing medical education system has been shown to contribute to improved quality of care and patient outcomes (Institute of Medicine, 2015).

Alternative effective educational strategies include providing access to online or print versions of medical genetics and genomics journals as well as textbooks containing the core knowledge and the latest advances in the field of genetics and genomics (Guttmacher et al., 2007). Due to the rapid pace at which genomics technology advances, educational strategies need to be designed in such a way so as to keep the workforce continually up-to-date in diagnostic and therapeutic measures, especially pertaining to pharmacogenomics and the use of tumor genomic data for precise molecular diagnosis of cancer, selecting targeted therapy, and monitoring of response to treatment (Slade et al., 2016).

Extensive barriers would first need to be overcome for the successful integration of the advances in genetic and genomic technologies into clinical care. In this regard, national healthcare system planners, administrators, and policy makers of LMICs would first need to establish collaborative ties and seek technical assistance from healthcare institutions abroad and from inter-governmental agencies such as the World Health Organization (Wonkam et al., 2010; Tekola-Ayele and Rotimi, 2015). This would enable them to overcome the existing lack of local human, technological, and financial resources. It would also empower them to advance genetic and genomic capacity building by fostering the development of genomic educational initiatives beginning from premedical education through medical specialty training to subspecialty training. Such collaborative efforts would help lay a solid foundation for building up the genomic literacy of the healthcare workforce in their respective countries within the context of their national economic and socio-cultural uniqueness.

## Author Contributions

NS gathered the literature data and wrote the manuscript. VD critically revised the manuscript for important intellectual content. Both authors read and approved the final manuscript.

## Conflict of Interest

The authors declare that the research was conducted in the absence of any commercial or financial relationships that could be construed as a potential conflict of interest.
